# The analysis of PIK3CA mutations in gastric carcinoma and metanalysis of literature suggest that exon-selectivity is a signature of cancer type

**DOI:** 10.1186/1756-9966-29-32

**Published:** 2010-04-16

**Authors:** Stefano Barbi, Ivana Cataldo, Giovanni De Manzoni, Samantha Bersani, Simona Lamba, Silvia Mattuzzi, Alberto Bardelli, Aldo Scarpa

**Affiliations:** 1 Department of Pathology, Section of Anatomic Pathology, University of Verona, Verona, Italy; 2 Department of Surgery, University of Verona, Verona, Italy; 3 Laboratory of Molecular Genetics, The Oncogenomics Center, Institute for Cancer Research and Treatment, University of Torino Medical School, Candiolo, Italy; 4 FIRC Institute of Molecular Oncology, Milan, Italy; 5 ARC-NET Center for Applied Research on Cancer, Verona, Italy

## Abstract

**Background:**

*PIK3CA *is one of the genes most frequently mutated in human cancers and it is a potential target for personalized therapy. The aim of this study was to assess the frequency and type of *PIK3CA *mutations in gastric carcinoma and compare them with their clinical pathological correlates.

**Methods:**

We analysed 264 gastric cancers, including 39 with microsatellite instability (MSI), for mutations in the two *PIK3CA *hotspots in exons 9 and 20 by direct sequencing of DNA obtained from microdissected cancer cells.

**Results:**

The cases harbouring mutations were 42 (16%). All were heterozygous missense single base substitutions; the most common was H1047R (26/42; 62%) in exon 20 and the second was Q546K (4/42; 9.5%) in exon 9. All the mutated MSI cases (8/39) carried the H1047R mutation. No other association between *PI3KCA *mutations and their clinical pathological covariates was found. A metanalysis of the mutations occurring in the same regions presented in 27 publications showed that ratio between exon 20 and exon 9 prevalences was 0.6 (95% CI: 0.5 -0.8) for colon, 1.6 (95% CI: 1.1 -2.3) for breast, 2.7 (95% CI: 1.6 -4.9) for gastric and 4.1 (95% CI: 1.9 -10.3) for endometrial cancer.

**Conclusions:**

The overall prevalence of *PIK3CA *mutations implies an important role for *PIK3CA *in gastric cancer. The lack of association with any clinical-pathological condition suggests that mutations in *PIK3CA *occur early in the development of cancer. The metanalysis showed that exon-selectivity is an important signature of cancer type reflecting different contexts in which tumours arise.

## Background

Gastric cancer is the second cancer cause of death in the world, although its incidence has declined in Western countries. Despite advances in its molecular characterization, to date, the only effective treatment is surgery with curative intent and the median 5-year survival is 25% [1].

Sporadic gastric cancer may arise along two major molecular pathways: one involves gross chromosomal alteration with multiple losses and gains of large chromosomal regions; the second is characterized by widespread somatic alterations in simple repetitive genomic sequences (microsatellites), as a result of defective DNA mismatch repair complex. These latter are defined microsatellite unstable tumors (MSI), represent about 15% of all the gastric tumors and are associated with a more favorable prognosis, larger size, female gender, advanced age, less lymph node involvement, intestinal histotype and antral location [2]. Common alterations found associated with MSI include promoter methylation of *MLH1 *[3] and mutations of *TGFBR2, IGFR2 *and *BAX *[4].

Microsatellite stable (MSS) gastric neoplasms show a different set of alterations: several proto-oncogenes, including *MET*, *FGFR2 *and *ERBB2*, are frequently amplified [5] while inactivation of both alleles of *TP53 *by loss of heterozygosity and mutation is the most frequent genetic event associated with MSS phenotype [6]. Moreover, loss of *TP73, APC*, *DCC*, *FHIT *and *TFF1 *are also frequently detected [5,7].

*PIK3CA *is a gene that encodes for the p110-alpha subunit of phosphoinositide-3-kinase (PI3K). Recently, a key role as oncogene is emerging for *PIK3CA*, as it is one of the genes most frequently hit by somatic mutations in several types of human cancer [8,9]. PI3K is part of a family of ser-thr-kinases that interacts with phosphatidylinositol bisphosphate (4,5-PIP2) to produce the phosphatidylinositol trisphosphate (3,4,5-PIP3), a second messenger with several functions. PIP3 mainly binds the plekstrine homology (PH) domain of a number of target molecules and leads to their activation through cell membrane targeting or modulation of their activity. One of the best characterized targets of PI3K lipid products is the protein kinase Akt. PI3K/Akt activation was demonstrated to be involved in the regulation of several cellular functions like cell survival, cell growth and angiogenesis stimulation, inhibition of apoptosis, translation of several proteins and hence, in the development of cancer [10,11].

Of the twenty exons that compose the *PIK3CA *gene, more than 75% of the mutations are found in two hot-spots located in exons 9 and 20, which encode for the helical and kinase domains, respectively [8]. Expression of the most common variants (E542K, E545K and H1047R) is associated with an increased lipid kinase activity and is oncogenic both in cell coltures and in vivo [12,13]. Mutations affecting the two hot-spots have recently been demonstrated to be functionally different [14] and their respective rates of mutation have been often reported as associated to specific cancer types or particular patient features [15,16].

In this study, we analysed 264 gastric cancers for the presence of mutations in the exons 9 and 20, by means of direct sequencing, and correlated the presence of mutations with clinical-pathological features, including MSI phenotype. In addition, we compared the prevalence of mutations occurring in the two exons with other studies investigating primary specimens of human cancer.

## Methods

### Patients and samples

Our study included 264 consecutive cases of advanced gastric cancer obtained from patients undergoing surgical intervention between 1989 and 2003 at the University of Verona. All patients were treated by radical surgical removal with resection margins free of microscopic disease and did not receive pre- or postoperative chemo- or radiotherapy. Histological classification was according to Laurén and the unified 1997 TNM system for gastric carcinoma was used for pathological staging. The clinical pathological features of the series are detailed in Table 1. This study was presented, reviewed and approved by the Local Ethics Committee of the Verona Hospitals Concern to include samples used for this analysis. Tumor samples were obtained with informed consent from the insititutions that provided the materials.

**Table 1:** Clinical features of 264 cases of gastric cancers analyzed for mutations in PI3KCA.

Parameter	Categories	Frequency
**Gender**	*F*	89 (33.7%)
	*M*	175 (66.3%)
		
**Age**	*mean (sd)*	67.4 (11.2)
		
**Lauren**	*Intestinal*	170 (65.4%)
	*Mixed*	27 (10.4%)
	*Diffuse*	63 (24.2%)
		
**pT**	*2*	99 (37.4%)
	*3*	129 (48.7%)
	*4*	36 (13.6%)
		
**pN**	*0*	53 (20.2%)
	*1*	100 (38.0%)
	*2*	80 (30.4%)
	*3*	30 (11.4%)
		
**pM**	*0*	215 (87.4%)
	*1*	31 (12.6%)
		
**Tumor Location**	*Antrum*	107 (40.5%)
	*Body*	72 (27.3%)
	*Fundus*	69 (26.1%)
	*Linitis*	12 (4.5%)
	*Gastric stump*	4 (1.5%)
		
**MSI**	*MSI*	39 (14.8%)
	*MSS*	225 (85.2%)

### Mutation analysis

Normal and tumor DNA was extracted from manually microdissected paraffin-embedded tissues as described [17]. Mononucleotide microsatellites *BAT25 *and *BAT26 *(located in introns of the *MSH2 *and *KIT *genes, respectively) were examined by PCR amplification using fluorescent dye-labeled primers as described [18]. PCR amplification and sequencing of *PIK3CA *exons 9 and 20 have been performed as described [19], using the following primers Exon9_Forward: GGGAAAAATATGACAAAGAAAGC; Exon9_Reverse: CTGAGATCAGCCAAATTCAGTT; Exon9_Sequencing: TAGCTAGAGACAATGAATTAAGGGAAA-3; Exon20_Forward: CTCAATGATGCTTGGCTCTG; Exon20_Reverse: TGGAATCCAGAGTGAGCTTTC; Exon20_Sequencing: TTGATGACATTGCATACATTCG.

Sequence differences from the NCBI reference sequence were identified via manual inspection of aligned electropherograms assisted by the Mutation Surveyor software package (SoftGenetics, State College, PA).

### Meta-analysis

To investigate the pattern of *PIK3CA *mutations in other studies involving gastric as well as other cancer types, we analysed the prevalence of *PIK3CA *mutations in data already present in literature and/or the COSMIC database [20]. The steps performed to search, select papers and collect data are detailed in Additional File 1. The full list of references of included studies is provided in Additional File 2.

### Statistical analysis

For meta-analysis, pooled prevalence estimates, prevalence ratios and confidence intervals were calculated using fixed-effects Poisson regression, adjusted for the number of cases analyzed per study. Prevalences and confidence intervals of single studies were evaluated using Clopper and Pearson method [21]. Correlation of the presence of the H1047R mutation with clinical-pathological features, p-values and confidence intervals were evaluated by means of logistic regression analysis. Correlation with survival was evaluated by means of log-rank test. For Cox multivariate regression, we selected the most informative variables among the models that included mutational status, using a 'forward' stepwise method. A p-value less than 0.05 was considered significant. For all the calculations and illustrations the R statistical software package was used [22].

## Results

We analysed the sequences of exons 9 and 20 of the *PIK3CA *gene in 264 advanced gastric cancers. The list and frequency of mutations found are detailed in Table 2. A total of 42 cases (15.9%; 95% CI 11.7% - 20.9%) harbored at least one mutation in the regions analyzed. All the mutations found were heterozygous missense single base substitutions. The most common mutation was H1047R occurring at the active site of the kinasic domain in exon 20 and representing 62% of all the mutations. The second most common mutation was Q546K that involves an aminoacid change in the helicase domain in exon 9 and represents 9.5% of all the mutations found.

**Table 2:** Frequency of PI3KCA mutations found in 264 gastric cancers, by mutation type.

	Mutation	Overall frequency (MSI only)	Percent/total cases	Percent/mutated cases
***Exon 9***	*E542K*	2	0.76%	4.76%
	*E545K*	2	0.76%	4.76%
	*Q546K*	4	1.52%	9.52%
	
	Total Mutations (ex. 9)	8	3.03%	
				
***Exon 20***	*M1043V*	1	0.38%	2.38%
	*H1047R*	26 (8)	9.85%	61.90%
	*H1048T*	1	0.38%	2.38%
	*G1050D*	2	0.76%	4.76%
	*T1052I*	1	0.38%	2.38%
	*T1053I*	1	0.38%	2.38%
	*D1056N*	2	0.76%	4.76%
	*L1067F*	1	0.38%	2.38%
	
	Total Mutations (ex.20)	35	13.26%	
				
***Total Mutations***		42	15.91%	

We found two missense mutations namely T1052I and T1053I that were never reported before. The mutations were confirmed using a second pair of primers (see Additional File 1). Both mutations involve an aminoacidic change from threonin to isoleucin that implies a change in the hydrophobic properties of the residues and may potentially affect the protein function. One case harboured two mutations namely E545K and L1067F, in exons 9 and 20, respectively.

In our series, MSI cases only harbored the H1047R mutation. H1047R was, in fact, observed in 8 of 39 MSI cases and was significantly associated with MSI status (OR 3.0; 95% CI 1.0 - 7.9; Fisher's test *P *= 0.035). The presence of mutation H1047R did not correlate with either survival or other clinical pathological features generally associated with MSI, possibly due to the small number of cases harboring the mutation. Furthermore, we did not observe any significant association between the presence of mutation and survival when considering MSI cases only. In addition, we did not find any correlation with clinical pathological features when considering the presence of mutations in both exons (Table 3) or in exon 9 and 20 separately. A multivariate survival analysis was performed in order to evaluate the effect of the presence of mutation together with other clinical-pathologic variables (Table 4). After selection of the best model, TNM stage, age and tumor location were significantly associated with survival, whereas only a marginal effect was observed for MSI status.

**Table 3:** Distribution of Clinical-pathological covariates according to the presence of *PI3KCA *mutations in 264 gastric cancers.

Parameter	Categories	Wt	Mutated	Odds Ratio (95% CI)	P
**Gender**	*F*	74 (83.1%)	15 (16.9%)	1	0.766
	*M*	148 (84.6%)	27 (15.4%)	0.9 (0.5 - 1.8)	
					
**Age**	*mean*	67.47	66.81		0.771
					
**pT**	*2*	88 (88.9%)	11 (11.1%)	1	0.077
	*3*	108 (83.7%)	21 (16.3%)	1.6 (0.7 - 3.5)	
	*4*	26 (72.2%)	10 (27.8%)	3.1 (1.2 - 8.1)	
					
**pN**	*0*	42 (80.8%)	10 (19.2%)	1	0.840
	*1*	86 (86.0%)	14 (14.0%)	0.7 (0.3 - 1.7)	
	*2*	67 (83.8%)	13 (16.2%)	0.8 (0.3 - 2.1)	
	*3*	26 (86.7%)	4 (13.3%)	0.6 (0.2 - 2.2)	
					
**pM**	*0*	182 (85.0%)	32 (15.0%)	1	0.298
	*1*	24 (77.4%)	7 (22.6%)	1.7 (0.6 - 4.0)	
					
**Lauren**	*Intestinal*	147 (86.5%)	23 (13.5%)	1	0.275
	*Mixed*	22 (81.5%)	5 (18.5%)	1.5 (0.5 - 4.0)	
	*Diffuse*	49 (77.8%)	14 (22.2%)	1.8 (0.9 - 3.8)	
					
**Location**	*Antrum*	93 (86.9%)	14 (13.1%)	1	0.394
	*Body*	58 (79.5%)	15 (20.5%)	1.7 (0.8 - 3.9)	
	*Fundus*	59 (85.5%)	10 (14.5%)	1.1 (0.5 - 2.7)	
					
**Grading**	*G1*	13 (86.7%)	2 (13.3%)	1	0.652
	*G2*	76 (87.4%)	11 (12.6%)	0.9 (0.2 - 6.5)	
	*G3*	117 (83.0%)	24 (17.0%)	1.3 (0.3 - 8.9)	
					
**Microsatellite instability**	*MSI*	31 (79.5%)	8 (20.5%)	1	0.408
	*MSS*	191 (84.9%)	34 (15.1%)	0.7 (0.3 - 1.7)	
					
**Survival rate at 2 years (95% CI)**		46.7% (40.5%-53.9%)	46.9% (32.4%-67.8%)		0.941

**Table 4:** Multivariate Cox survival analysis of 245 gastric cancer patients.

Parameter	Category	HR (95% CI)	P-Value
**PI3KCA status**	*wt*	1.0	0.630
	*mutated*	1.1 (0.7-1.7)	
			
**Stage**	*I*	1.0	<0.001
	*II*	3.1 (1.1-9.1)	
	*III*	11.6 (4.2-31.8)	
	*IV*	19.1 (6.8- 53.2)	
			
**Age (10 years increment)**		1.3 (1.1-1.5)	<0.001
			
**Tumor Location**	*Antrum*	1.0	0.004
	*Body*	1.1 (0.7-1.5)	
	*Fundus*	1.8 (1.3-2.6)	
			
**MSI status**	*MSI*	1.0	0.077
	*MSS*	1.7 (0.9-3.0)	

In order to systematically compare our results with the available literature for stomach and other cancer types, we selected 38 series described in 27 papers analyzing mutations in the *PIK3CA *locus in primary cancer samples (the full list of references is provided in Additional File 2). We limited the analysis to the mutations occurring at the aminoacids 542-549 and 1043-1048, of exons 9 and 20, respectively, that were analyzed in common between the series. These regions contain the large majority of mutations observed in *PIK3CA *[8].

**Figure 1:**
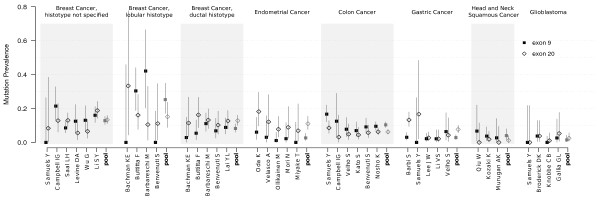
**Point and 95% confidence interval estimates of prevalence of mutations affecting exon 9 and 20 of *PI3KCA *in 36 series**. Mutations affecting exon 9 and 20 are shown as solid filled boxes and empty diamonds, respectively. The pooled estimates for each group are shown in grey.

The prevalence of mutations in exons 9 and 20 for each series is represented in Figure 1. Although the overall rates of mutation was variable among the series, even of the same cancer type, the rates of mutation in exon 9 and 20 significantly correlated to each other (Spearman's ρ = 0.75, P-value < 0.001), suggesting that the ratio between exon 9 and exon 20 mutations is dependent on the cancer type. Therefore, we evaluated the pooled ratio of prevalence between exon 20 and 9 in different studies grouped by cancer type, by means of Poisson regression analysis. Results are shown in Table 5. For breast cancer, given the large number of studies reported, we divided the series according to the histotype (ductal and lobular), where the information was available, and categorized the remainder series as breast cancer with histotype unspecified. Among series of ductal histotype, prevalence of mutations was significantly biased towards exon 20, whereas a marginally significant preference for exon 9 was observed for lobular histotype series (see Table 5 and Figure 1). The studies on colon cancer showed a significantly increased prevalence of mutations in exon 9 with all the series having a similar mutational pattern. Tumors of the endometrium were significantly more hit by mutations affecting exon 20. For gastric cancer, the present series as well as the series reported by Samuels showed a greater prevalence of exon 20, whereas the remainder series showed little or no difference between exons.

**Table 5:** Overall frequency and pooled prevalence ratio of mutations affecting the two hot spots of *PIK3CA *located in Exon 9 and exon 20 in 36 series grouped by cancer type

Tumor Type	nr. series	total cases	Exon 9	Exon 20	Ex20/Ex9 Prevalence Ratio (95% CI)	P-value
*Breast Cancer (histotype not specified)*	6	788	101	105	1.0 (0.8 -1.4)	0.7805
*Breast Cancer (lobular histotype)*	4	99	25	15	0.6 (0.3 -1.1)	0.1178
*Breast Cancer (ductal histotype)*	5	499	41	64	1.6 (1.1 -2.3)	**0.0260**
*Endometrial Cancer*	5	263	7	29	4.1 (1.9 -10.3)	**0.0007**
*Colon Cancer*	6	1292	134	80	0.6 (0.5 -0.8)	**0.0003**
*Gastric Cancer*	5	602	17	46	2.7 (1.6 -4.9)	**0.0005**
*Head and Neck squamous Cancer*	3	175	7	2	0.3 (0.0 -1.2)	0.1182
*Glioblastoma*	4	203	3	5	1.7 (0.4 -8.1)	0.4842

## Discussion

The aim of this study was to characterize the mutational status of *PIK3CA *in a large series of gastric cancers in order to determine its prevalence with an adequate precision and to correlate it with clinical-pathological features. The overall prevalence of mutations was 15.9%, a value that is within the range of the currently available literature [8,23-25], nonetheless the prevalences observed in different series are heterogeneous, ranging from 4.5% to 25%. Reasons for such a heterogeneity may be due to specific interactions of the mutations with environmental and genetic backgrounds, although experimental factors can not be excluded.

To our knowledge, the mutations involving aminoacids 1052 and 1053 (T1052I and T1053I) were never published before, nor are described in the COSMIC database, despite the large number of studies investigating the region. As the mutations imply a change in the hydrophobicity of the aminoacidic residue, a functional role cannot be excluded. The mutations were found in MSS cases that did not show any particular feature.

We also found that the most common *PIK3CA *mutation (H1047R) was significantly associated with MSI phenotype. The association is moderate and would benefit from confirmation on an indipendent series. An association between *PIK3CA *mutations and MSI has been reported or at least suggested in both colon and stomach cancer [8,23,24,26]. At variance with our findings, in the two studies regarding gastric cancer and reporting mutations by MSI status, exon 9 and exon 20 mutations were evenly distributed between the subtypes [23,24]. However, the small number of mutated MSI cases prevents statistical comparison. The fact that only one type of mutation was found in our series of MSI tumors is not surprising as the narrow spectrum of alterations of MSI gastric tumors may, in turn, restrict the type of *PIK3CA *mutations that are oncogenic in that context.

Despite the large series analyzed, we did not find any correlation of *PIK3CA *mutations with clinical pathological features of gastric cancers apart from the association between MSI and H1047R. The lack of associations suggests that alteration of *PIK3CA *is an event that occurs early in a subset of gastric cancers that progresses towards malignancy through other mechanisms. In fact, in a multivariate survival model there was no evident effect of the presence of mutation on prognosis.

Based on our meta-analysis, the ratio between mutation prevalences in exons 9 and 20 can be generally considered a signature of cancer type. In particular, we found a significant exon bias for colon cancer, breast cancer with ductal histotype and endometrium cancer. In colon cancer, exon 9 is significantly more hit than exon 20. This confirms suggestions from previous studies [8,23,27]. The opposite mutational pattern was consistently found in studies regarding endometrial cancer with exon 20 largely more hit than exon 9. This peculiarity was already pointed out and suggests a specific mechanism of *PI3KCA *involvement for endometrial cancer [28-30].

It is less clear whether an exon bias exists in breast cancer as many studies are apparently contradictory (see Figure 1). However, for studies that did furnish the information about the histotype of each sample, we observed a different exon preference between lobular and ductal histotypes as already suggested [15]. For ductal histotype, exon 20 was significantly more hit compared to exon 9, whereas a slight but inverse tendency was found in series of lobular breast cancers. This pattern is not evident in studies where the information about histotype is not available, possibly as an result of mixing different kinds of tumours together.

For squamous head and neck tumors a slight tendency to a greater prevalence of exon 9 mutations was observed among series, although the low incidence of *PIK3CA *mutations in this kind of neoplasm has probably prevented this tendency to stand out. For glioblastoma, there was no evidence of exon-selectivity, due to the fact that a high percent of non hot-spot mutations are frequently found in this disease [8,31].

Finally, in stomach cancer series, exon 20 resulted to be more involved than exon 9, although a common trend among the series was substantially missing. The heterogeneity in both overall prevalence and exon-selectivity in stomach cancer may be due to the strong influence that specific etio-pathologic, genetic and environmental factors have on this disease.

Although several of the observations presented in our meta-analysis were sporadically suggested or demonstrated in single papers, this approach allows to gather more convincing evidences by pooling similar studies. Moreover, the meta-analysis has the further advantage of providing an outlook and an estimate of *PIK3CA *exon-selectivity and standardized rate of mutation in different cancer types, although this might be affected by the limitations derived from retrospective studies.

The association of specific mutations with either cancer type or subtype is in line with recent findings about different mechanisms through which these mutations exert their oncogenic potential. In fact, it has been shown that mutations occurring at the kinasic domain are dependent upon binding with p85, another component of PI3K, to be fully oncogenic, whereas mutations in the helical domain are dependent upon RAS-GTP binding [14]. The dependence of *PIK3CA *mutations on other signalling components is in keeping with the fact that the genetic background in which tumours develop may require and select specific altered activities of p110-alpha.

## Conclusions

We found a relatively high prevalence of *PIK3CA *somatic mutations further supporting the role of *PIK3CA *as a major oncogene in gastric cancer. Such prevalence was highly biased towards exon 20, in particular, in MSI cases which seem to carry only one type of exon 20 mutations. By analysis of the mutations occurring in the two standard hot-spot regions of *PIK3CA *in 27 published papers on six major cancer types (colorectal, breast ductal, breast lobular, stomach, endometrium, head and neck and glioblastoma), we found that exon-selectivity is an important signature of cancer type and subtype reflecting different contexts in which tumours arise.

## Competing interests

The authors declare that they have no competing interests.

## Authors' contributions

SB performed data analysis and manuscript drafting; IC partecipated in manuscript drafting and revising; GDM contributed to conception and design, collected specimens and provided clinical informations; SB performed microdissection and DNA purification and carried out microsatellite analysis; SL and SM performed *PI3KCA *mutation analysis; AB contributed to conception and design of experiments and supervised molecular analysis; AS contributed to conception and design of experiments and approved the final version of the manuscript. All authors read and approved the final manuscript.

## Supplementary Material

Additional file 1 **Supplementary Material and Methods**. Supplementary Material and Methods Click here for file

Additional file 2 **Metanalysis references**. List of papers analyzed for the presence of *PI3KCA *mutations Click here for file
