# Supplementary material for: The analysis of PIK3CA mutations in gastric carcinoma and metanalysis of literature suggest that exon-selectivity is a signature of cancer type
Source: J Exp Clin Cancer Res. 2010 Apr 16;29(1):32. doi: 10.1186/1756-9966-29-32 (PMC2865450; doi:10.1186/1756-9966-29-32)
Supplement: Additional file 1 — Supplementary Material and Methods. Supplementary Material and Methods [file 1756-9966-29-32-S1.PDF]

## Supplementary methods

### Mutation analysis

The confirmatory PCR on exon 20 were performed using the following primers:

ex20_f ACATTCGAAAGACCCTAGCC; ex20_r TTTTCAGTTCAATGCATGCTG.

### Metanalysis

### Search strategy

We selected studies published up to September 2008, analyzing the sequence of hot-spots of the *PI3KCA* locus in primary cancer specimens, by either Sanger sequencing or pyrosequencing. The studies search, selection and data extraction have been performed and revised by both SB and IC, who are a bionformatician and a pathologist, respectively.

The primary source for searching eligible studies was the COSMIC database, a repository which collects information about reported mutations per locus in published papers identified by the Pubmed identifier. The list of mutated *PIK3CA* samples can be obtained through the following URL:

http://www.sanger.ac.uk/perl/genetics/CGP/cosmic_export/pik3ca_1-1069?action=exported&neg=off&refs=off&ln=PIK3CA&start=1&end=1069&coords=AA%3AAA&format=tsv&x=40&y=12

From the subset of the table including only mutations affecting the two hot-spots considered in this analysis and we extracted a first set of candidate papers.

In addition, the following search key was used to query the Pubmed website: ("MUTATIONS"[Title/Abstract] OR "MUTATION"[Title/Abstract]) AND ("1"[Publication Date] : "2008/09/31"[Publication Date]) AND PIK3CA[TITLE]. The abstracts resulting from this second query were individually analyzed for relevance.

**Inclusion/exclusion criteria**

We included only studies for which the information about site and type of mutation, and sample cancer type could be extracted either from the COSMIC database or from the paper. In addition, as the purpose of this analysis was to aggregate studies investigating series with homogenous patient features, we excluded studies with particular patient selections that were not replicated by other investigators. The same criteria were applied at series level in multi-series studies. That is, we excluded part of the patients reported in the original paper when these were not matched by at least another similar study. Furthermore, we excluded studies regarding lung cancer because the rarity of *PIK3CA* mutations affecting this cancer type, would have added little or no information as far as prevalence ratio of mutations at the two *PIK3CA* hot-spots is concerned. Finally, we required that both hot-spots of *PI3KCA* had been sequenced for each study. At sample level, we exluded data on cell lines.

**Data extraction**

For extraction of data, namely mutation type, site and sample features we used the COSMIC database as the primary source, or the paper when the information was not available in COSMIC. The total number of primary cancer analyzeds was retrieved from individual papers. The data were aggregated by cancer type and mutation site. Due to the large number of studies published on breast cancer, we found more convenient to aggregate data by histotype, as this was available in several studies.

No system to quantitatively weigh the quality of individual studies was adopted and no study was dropped due to quality issues. We made no effort to reduce heterogeneity within groups, due to f the low number of studies per group and also due to the fact that, in this particular analysis, heterogeneity was considered part of the results and is discussed in the paper.

The full list of references of studies included is provided in Supplementary Table 2.
